# Prevalence and Social Determinants of Smoking in 15 Countries from North Africa, Central and Western Asia, Latin America and Caribbean: Secondary Data Analyses of Demographic and Health Surveys

**DOI:** 10.1371/journal.pone.0130104

**Published:** 2015-07-01

**Authors:** Chandrashekhar T. Sreeramareddy, Pranil Man Singh Pradhan

**Affiliations:** 1 Department of Population Medicine, Faculty of Medicine and Health Sciences, University Tunku Abdul Rahman, Bandar Sungai Long, Selangor, Malaysia; 2 Department of Community Health Sciences, Patan Academy of Health Sciences f, Lalitpur, Nepal; Hunter College, UNITED STATES

## Abstract

**Background:**

Article 20 of the World Health Organisation Framework Convention on Tobacco Control calls for a cross-country surveillance of tobacco use through population-based surveys. We aimed to provide country-level prevalence estimates for current smoking and current smokeless tobacco use and to assess social determinants of smoking.

**Methods:**

Data from Demographic and Health Surveys done between 2005 and 2012, among men and women from nine North African, Central and West Asian countries and six Latin American and Caribbean countries were analyzed. Weighted country-level prevalence rates were estimated for ‘current smoking’ and ‘current use of smokeless tobacco (SLT) products’ among men and women. In each country, social determinants of smoking among men and women were assessed by binary logistic regression analyses by including men's and women's sampling weights to account for the complex survey design.

**Findings:**

Prevalence of smoking among men was higher than 40% in Armenia (63.1%), Moldova (51.1%), Ukraine (52%), Azerbaijan (49.8 %), Kyrgyz Republic (44.3 %) and Albania (42.52%) but the prevalence of smoking among women was less than 10% in most countries except Ukraine (14.81%) and Jordan (17.96%). The prevalence of smokeless tobacco use among men and women was less than 5% in all countries except among men in the Kyrgyz Republic (10.6 %). Smoking was associated with older age, lower education and poverty among men and higher education and higher wealth among women. Smoking among both men and women was associated with unskilled work, living in urban areas and being single.

**Conclusion:**

Smoking among men was very high in Central and West Asian countries. Social pattern of smoking among women that was different from men in education and wealth should be considered while formulating tobacco control policies in some Central and West Asian countries.

## Background

In 2010, globally, an estimated 34·5 million deaths [1] and 54% of Disability adjusted Life Years (DALYS) were caused by non-communicable diseases (NCDs)[2]. Tobacco smoking, including second-hand smoke is a leading risk factor for global disease burden [3], and accounts for 6.3% of global DALYS [2]. Despite a decreasing trend in smoking rates among men and women worldwide an estimated 967 million smokers lived in 187 countries as of year 2012[4]. Tobacco is also consumed in smokeless forms in South and Southeast Asian countries [5,6]. A global tobacco control treaty Framework Convention on Tobacco Control (FCTC), launched by World Health Organization (WHO) in 2005 is the first modern-day global public health treaty ratified by 178 countries to-date [7]. The WHO FCTC is a global response to the tobacco epidemic and calls on countries to set out specific steps related to taxes, pricing, banning advertisements, creating smoke free spaces, health warnings on packages and combating illicit trade. The article 20 of the WHO FCTC also recommends a cross-country surveillance of tobacco use through population-based surveys [8]. In this direction, multinational surveys such as Global Adult Tobacco Survey (GATS)[9], World Health Surveys (WHS)[10] and International Tobacco Control (ITC) policy evaluation projects [11], WHO STEPS program [12] were undertaken. Information from these projects is important for monitoring the progress of tobacco control efforts and formulating new control strategies.

Literature about tobacco use in Latin American and Caribbean countries is limited. Some countries of these regions are covered in multinational surveys such as GATS [9],WHS [10], ITC projects [11] but comprehensive data on all countries is unavailable for entire regions. Literature on tobacco use is available for eight countries of the former Soviet Union [13], four Latin American countries [14], a few surveys in other countries [15–17], reviews about the Caribbean countries [18] and Latin America [18,19]. However, available literature may not accurately reflect the tobacco use estimates, the scale of the tobacco epidemic, its social pattern and types of tobacco products consumed in these regions due to heterogeneous survey designs, and inconsistent definitions of smoking. Demographic and Health Surveys (DHSs) done on nationally representative samples of men and women have provided national-level estimates of smoking and smokeless tobacco (SLT) use for each country, studied the social distribution of tobacco use, and type of tobacco products consumed in nine South and Southeast Asian countries [6] and for 30 Sub Saharan African countries [20]. In this report, we provide prevalence estimates of smoking and SLT use in the adult populations of 15 countries from the regions of North Africa, Central and West, Asia Latin America and the Caribbean. We also explore the social factors associated with smoking in these populations.

## Methods

### Ethics statement

Since there was no identifiable information about the participants collected during the survey or data archived by DHS, a separate institutional ethical clearance approval was not required to prepare this report. The DHS survey protocols were approved by institutional review boards of ICF, the DHS program and the local institutions of each participating country. Before each interview, the respondents were informed about the details of the survey, voluntary participation, and confidentiality of information and informed consent was obtained.

### Data source

We used the data from most recent DHS done between 2005 and 2012 in which information about tobacco use was collected for male and/or female respondents. The original datasets of DHSs in 15 countries of North Africa, Latin America, Central and West Asia were obtained from the DHS program website (http://www.dhsprogram.com/) with their written permission. DHSs are large, nationally representative, cross-sectional, house-to-house surveys conducted by in-country/local institutions with funding from the United States Agency for International Development (USAID) and technical assistance from ORC (Opinion Research Corporation) Macro International Inc. Calverton, Maryland, USA. Trained interviewers conducted the health interviews of eligible men and women using standardized core questionnaires enabling cross-country comparisons. In general, DHSs adopt two-stage, stratified cluster sampling design, wherein clusters from both urban and rural areas are selected by probability proportional to size technique followed by a random selection of households from within the selected clusters. In some countries, DHS oversamples lesser populated provinces or regions. In each selected household, the head of the household responds to all general questions related to the household and lists all the members who are usual residents of the household. All eligible men and women were interviewed by trained interviewers in a local language. All interviews were supervised to ensure adherence to guidelines, quality control and minimizing non-response [21,22].

### Outcome variable

In men's and women's questionnaires, four main questions were asked about tobacco use. The questions were similar in structure across the countries, but for some exceptions in response options in some countries. The respondents were asked to provide 'yes' or 'no' response to the first two questions. For the third question, country-specific options on the type of tobacco use were provided. For example, snuff in Haiti. The general outline of the tobacco use question is given below.

1. Do you currently smoke cigarettes?
2. Do you currently smoke or use any other type of tobacco?
3. What (other) type of tobacco do you currently smoke or use? (pipe, chewing tobacco, snuff, etc.)
4. In the last 24 hours, how many cigarettes did you smoke? (response as numerical)

Similar to our previous reports for South and Southeast Asia [6] and Sub Saharan Africa regions [20], we constructed two outcome variables, namely 'current smoking' and 'current SLT use' based on the responses provided to the four questions.

#### Social variables

To study the social distribution of tobacco use, we used age (in single years) recoded as 15–24, 25–34, 35–44 and ≥45; marital status (classified as 'not in union', 'married', 'cohabiting partner' and 'single' which includes separated, widowed and divorced), place of residence (classified as 'rural' and 'urban'), current occupation ('unemployed', 'professional', 'agriculture' and 'unskilled/manual work'), educational level ('no education', 'primary', 'secondary' and 'higher') and household wealth index. Wealth index is a reliable proxy indicator for economic status and was calculated based on a standard set of household assets, dwelling characteristics and ownership of consumer items as observed by the interviewer [23]. Each household was classified into quintiles where first quintile was the poorest 20% of the households and fifth quintile was the wealthiest 20% of the households [24]. Religion was not included among the social factors since the information on religion was not collected in some countries, while in others >90% of the respondents were either Christians or Muslims.

### Statistical analysis

For each country, we estimated overall country-level weighted prevalence rates and their 95% CIs for ‘current smoking’ and ‘current SLT use’ separately for men and women. For SLT use, prevalence rates by social factors and multivariate analyses was not done since prevalence rates were very low in most countries and the data on SLT use was not available for women in eight countries. Weighted prevalence rates of smoking were calculated according to social factors. Binary logistic regression analyses were done to assess the association between smoking and social factors. To account for the complex sampling design adopted in DHS, men's and women's sampling weights, primary sampling units, sample stratum numbers were included in the analysis under *'svy'* command in Stata/IC version 10. Adjusted odds ratios (aOR), their 95% confidence intervals and p-values were calculated.

## Results

### Sample characteristics

Overall, the response rates were above 90% in all surveyed countries except Ukraine, Dominican Republic and Guyana. Tobacco use data were not collected from women in Azerbaijan and from men in Bolivia (Table 1). In most countries, nearly a third or more of the participants were aged 15–24 years, except Moldovan men and women in Ukraine, Egypt and Jordan. About two-third of the respondents were from urban areas in Armenia, Jordan, and Ukraine, but in Honduras, Guyana, Haiti, and Kyrgyz Republic two-thirds of them were from rural areas. In the remaining countries, the proportion of urban respondents was slightly higher. Nearly half or more of the respondents were either married or had a cohabiting partner and were educated up to secondary or higher level. In most countries, the percentage of respondents without any education was <10% except Egypt and Haiti. The distribution of the participants across the wealth quintiles was nearly uniform. The highest proportion of men was unskilled workers in all countries except Honduras and Haiti (agriculture was the highest) whereas in all countries highest proportion of women were unemployed (S1, S2 and S3 Tables).

**Table 1:** Survey characteristics, sample sizes and responses of Demographic and Health Surveys.

Country	Dates of fieldwork	Household sample	Women’s sample	Men’s sample	Response rate (%)
**1. Albania**	October 2008–April 2009	7999	7584	3013	96.0
**2. Armenia**	October 2010–December 2010	6700	5922	1584	93.0
**3. Azerbaijan**	July 2006–November 2006	7180	8444	2558	95.5
**4. Kyrgyz Republic**	August 2012–December 2012	8040	8208	2413	98.5
**5. Jordan** *	September 2012–December 2012	15190	11352	-	94.0
**6. Tajikistan** *	July 2012–September 2012	6432	9656	-	97.4
**7. Egypt** *	April 2005–June 2005	21972	19474	-	98.5
**8. Moldova**	June 2005–August 2005	11095	7440	2508	90.5
**9. Ukraine**	July 2007–November 2007	13379	6841	3178	87.5
**10. Bolivia‡**	February 2008–June 2008	19564	16939	6054	94.8
**11. Dominican Republic**	March 2007–August 2007	32431	27195	27975	89.5
**12. Honduras**	September 2011–July 2012	21362	22757	7120	91.7
**13. Peru** *	March 2012–December 2012	27218	23888	-	96.3
**14. Guyana**	March 2009–July 2009	5632	4996	3522	84.0
**15. Haiti**	January 2012–June 2012	13181	14287	9493	98.4

* men were not interviewed in these countries,

^‡^ men were not asked about tobacco use

### Prevalence of smoking and SLT use

Prevalence of smoking among men was higher than 40% in most countries of Central and West Asia, Armenia being the highest (63.1%). Among the Latin American and Caribbean countries, the prevalence of male smoking was highest in Guyana (30.5%) followed by Honduras (24.3%). Prevalence of SLT use among men was <5.0% in all the countries except for Kyrgyz Republic (10.6%) (Table 2). The prevalence of female smoking was lower than the male smoking in all the North African, Central and West Asian countries, but the highest female smoking prevalence was in Jordan and Ukraine (18.0% and 14.8% respectively). In Latin American and Caribbean, the prevalence of female smoking was highest in Bolivia (8.7%) followed by the Dominican Republic (6.4%). Female SLT use was lower than 5% all the countries, the highest being 3.2% in Haiti (Table 2). Tobacco users mostly smoked cigarettes in all the countries. The exceptions were the women from Egypt (44% smoked pipe and 38% chewed tobacco), women and men from Haiti (52% and 18% respectively were snuff users) (S1 and S2 Figs).

**Table 2:** Weighted prevalence estimates (95% confidence intervals) of smoking (cigarettes, pipe and others) and smokeless tobacco use (chewing, snuff) among men and women.

	Men		Women	
Country (survey year)	Smoking	SLT use	Smoking	SLT use
**North Africa, Central and West Asia**				
**1. Albania(2008–2009)**	42.52 (40.39, 44.65)	1.64 (0.98, 2.30)	4.18 (3.02, 5.33)	⁺
**2. Armenia (2010)**	63.06 (60.08, 66.04)	0.09 (-0.09, 0.27)	1.78 (1.14, 2.41)	⁺
**3. Azerbaijan (2006)**	49.80 (47.38, 52.21)	0.24 (0.10, 0.38)	-	⁺
**4. Kyrgyz Republic (2012)**	44.33 (41.86, 46.79)	10.60 (8.98,12.23)	2.76 (2.14, 3.38)	0.04 (-0.01, 0.09)
**5. Tajikistan (2012)** *	-	-	0.19 (0.07, 0.31)	0.03 (0.00, 0.07)
**6. Moldova (2005)**	51.06 (48.54, 53.57)	0.11 (-0.02, 0.24)	7.12 (6.41, 7.83)	0.02 (-0.01, 0.05)
**7. Ukraine (2007)**	52.00 (48.97, 55.02)	0.25 (0.07, 0.43)	14.81 (13.26,16.35)	⁺
**8. Jordan (2012)** *	-	-	17.96 (16.37,19.55)	⁺
**9. Egypt (2005)** *	-	-	0.60 (0.46, 0.74)	0.03 (0.00, 0.07)
**Latin America and Caribbean**				
**10. Bolivia (2008) ‡**	-	-	8.66 (7.99, 9.32)	⁺
**11. Dominican Republic (2007)**	11.50 (10.85, 12.14)	2.22 (1.93, 2.52)	6.39 (5.94, 6.85)	0.33 (0.24, 0.42)
**12. Honduras (2011–2012)**	24.28 (22.83, 25.73)	0.67 (0.43, 0.90)	1.74 (1.49, 1.99)	0.03 (0.01, 0.06)
**13. Peru (2012)** *	-	-	4.92 (4.34, 5.49)	⁺
**14. Guyana (2009)**	30.46 (28.11, 32.82)	0.54 (0.17, 0.91)	3.30 (2.60, 4.00)	⁺
**15. Haiti (2012)**	11.48 (10.49, 12.47)	3.13 (2.45, 3.82)	2.17 (1.83, 2.50)	3.15 (2.59, 3.71)

*Men were not interviewed,

^‡^ men were not asked about tobacco use,

^¶^ women were not asked about tobacco use,

^⁺^questions about smokeless tobacco use were not asked,

^-^ data was not available

## Distribution of Smoking and Its Association with Socio-Demographic Factors

### Urban-rural distribution

Distribution of smoking by social-demographic factors are shown in Tables 3–5, while the associations between smoking and social factors, by logistic regression analyses are shown in Tables 6 and 7. Urban-rural differences in male smoking rates were very small in most countries except Kyrgyz Republic (urban, 52.0 vs. rural, 40.6) and Guyana (urban, 24.2 vs. rural, 32.8) (Table 3) whereas female smoking rates were much higher in urban areas in all countries except Dominican Republic (Tables 4 and 5). Rural men were less likely to smoke in Ukraine (aOR 0.72, 95% CI 0.53, 0.97), Moldova (aOR 0.75, 95% CI 0.57, 0.99) and Honduras (aOR 0.73, 95% CI 0.60, 0.0.89) whereas rural women were less likely to smoke in most countries except Kyrgyz Republic, Tajikistan, Dominican Republic, and Peru (Tables 6 and 7).

**Table 3:** Weighted prevalence estimates (%) and 95% CIs of smoking by social factors among men in 10 countries (Central and West Asia, Latin America and Caribbean).

	Albania	Armenia	Azerbaijan	Kyrgyz Republic	Moldova	Ukraine	Dominican Republic⁺	Honduras	Guyana	Haiti
Domicile										
Urban	42.35(39.65, 45.05)	64.30(60.49, 68.12)	50.00(46.48, 53.52)	52.03(47.57, 56.50)	48.79(46.08, 51.50)	51.46(47.47, 55.44)	10.84(9.99, 11.68)	24.37(22.00, 26.75)	24.18(20.96, 27.39)	10.10(8.65, 11.55)
Rural	42.67(39.47, 45.87)	61.02(56.25, 65.79)	49.52(46.41, 52.63)	40.64(37.76, 43.51)	52.70(48.85, 56.55)	53.35(49.80, 56.91)	12.95(12.07, 13.83)	24.19(22.49, 25.88)	32.79(29.87, 35.72)	12.57(11.21, 13.93)
Age groups (years)										
15–24	23.50(19.88, 27.11)	40.75(34.36, 47.14)	22.27(18.36, 26.18)	16.38(12.99, 19.77)	40.74(36.81, 44.67)	35.83(31.66, 40.01)	4.23(3.58, 4.88)	23.18(20.75, 25.60)	17.30(14.02, 20.58)	4.34(3.37, 5.31)
25–34	55.24(50.28, 60.21)	73.36(68.84, 77.88)	60.99(56.34, 65.64)	49.90(45.74, 54.06)	60.30(55.50, 65.11)	55.80(51.64, 59.97)	10.08(8.73, 11.42)	27.65(24.99, 30.30)	31.31(27.14, 35.49)	12.71(10.71, 14.70)
35–44	53.72(50.02, 57.43)	74.03(68.07, 79.99)	64.46(59.85, 69.08)	66.43(62.01, 70.86)	57.39(52.87, 61.91)	58.04(53.41, 62.68)	17.58(15.98, 19.18)	22.12(19.37, 24.86)	41.94(37.81, 46.07)	16.20(13.86, 18.54)
45–59	49.13(44.24, 54.01)	76.31(68.26, 84.37)	57.50(52.08, 62.92)	67.47(60.85, 74.09)	50.74(46.54, 54.94)	63.79(58.29, 69.28)	19.57(17.94, 21.20)	23.99(20.88, 27.11)	41.07(34.72, 47.41)	21.20(18.44, 23.75)
Marital status										
Never in union	28.21(25.33, 31.09)	47.70(42.98, 52.42)	28.45(24.54, 32.35)	19.08(15.25, 22.92)	40.56(36.73, 44.39)	39.44(35.53, 43.35)	4.84(4.17, 5.51)	20.03(17.54, 22.52)	17.71(14.85, 20.56)	4.29(3.47, 5.11)
Married	52.78(50.04, 55.52)	75.02(71.03, 79.00)	60.32(57.62, 63.02)	58.34(54.76, 61.93)	53.48(50.47, 56.50)	55.96(52.26, 59.67)	8.27(7.00, 9.55)	14.38(12.09, 16.66)	27.44(24.36, 30.53)	16.67(14.89, 18.45)
Cohabiting partner	-	-	-	-	63.61(52.05, 75.18)	52.73(41.57, 63.90)	15.37(14.27, 16.47)	30.91(28.38, 33.43)	48.56(43.98, 53.13)	20.66(14.83, 26.49)
Single	68.70(54.83, 82.58)	80.51(69.92, 91.10)	66.88(51.62, 82.15)	63.59(50.57, 76.61)	74.17(66.67, 81.67)	71.85(65.30, 78.40)	22.41(20.30, 24.53)	41.37(36.27, 46.48)	54.13(47.18, 61.08)	27.23(22.91, 31.56)
Education										
No education	39.71(9.15, 70.27)	-	55.39(24.15, 86.63)	-	56.49(0, 116.73)	-	22.83(20.11, 25.54)	30.09(24.53, 35.65)	48.89(33.84, 63.95)	24.09(20.99, 27.20)
primary	48.59(44.71, 52.47)	55.88(47.71, 64.05)	41.75(11.82, 71.68)	17.94(0, 45.29)	44.55(17.35, 71.74)	79.70(39.78, 119.62)	14.90(13.92, 15.89)	26.80(25.05, 28.55)	43.31(38.66, 47.96)	11.39(9.93, 12.85)
Secondary	38.24(35.12, 41.36)	67.40(62.43, 72.36)	51.89(49.38, 54.41)	40.50(37.53, 43.47)	53.31(50.50, 56.13)	57.90(54.56, 61.23)	7.63(6.71, 8.55)	19.63(17.05, 22.22)	28.34(26.03, 30.64)	8.43(7.07, 9.78)
Higher	38.92(31.92, 45.92)	61.41(57.54, 65.29)	41.87(36.50, 47.23)	50.82(46.77, 54.87)	39.51(35.03, 43.99)	45.77(41.55, 49.98)	6.79(5.55, 8.02)	19.46(13.17, 25.74)	13.42(8.60, 18.23)	7.23(4.82, 9.64)
Wealth Index										
Poorest	45.08(39.76, 50.39)	55.92(49.02, 62.82)	58.27(53.25, 63.30)	38.41(34.23, 42.60)	60.02(53.87, 66.17)	61.64(55.88, 67.41)	16.98(15.80, 18.16)	28.75(26.00, 31.49)	52.56(47.59, 57.53)	14.72(12.89, 16.54)
Poorer	43.44(36.38, 50.50)	67.45(62.02, 72.88)	45.49(39.99, 50.99)	33.46(28.61, 38.30)	56.53(50.59, 62.47)	53.68(49.11, 58.25)	13.72(12.23, 15.21)	27.64(24.79, 30.49)	33.96(29.95, 37.97)	11.23(9.29, 13.17)
Middle	41.02(35.99, 46.05)	63.62(55.61, 71.64)	50.29(45.21, 55.37)	41.99(37.26, 46.73)	49.07(43.86, 54.28)	55.41(50.59, 60.23)	9.42(8.16, 10.67)	25.90(22.99, 28.81)	26.25(22.25, 30.24)	13.66(11.35, 15.97)
Richer	44.56(40.39, 48.74)	62.90(56.30, 69.51)	50.01(44.44, 55.59)	50.18(45.22, 55.14)	46.64(42.10, 51.17)	51.79(45.71, 57.86)	7.75(6.61, 8.89)	17.94(15.20, 20.69)	24.55(20.34, 28.76)	10.85(8.48, 13.22)
Richest	39.38(35.35, 43.40)	66.13(58.96, 73.29)	46.31(41.15, 51.48)	57.49(51.71, 63.26)	45.35(41.23, 49.47)	43.35(36.72, 49.99)	9.11(7.64, 10.57)	21.20(17.39, 25.01)	17.08(13.63, 20.53)	7.85(6.31, 9.40)
Occupation⁺										
Unemployed	21.21(17.49, 24.94)	38.80(32.19, 45.41)	29.00(24.61, 33.40)	11.13(7.17, 15.10)	43.59(39.67, 47.50)	30.58(25.96, 35.20)	-	13.44(7.40, 19.48)	9.48(5.81, 13.16)	4.23(3.12, 5.35)
Professional	45.80(41.09, 50.51)	65.18(58.19, 72.18)	48.02(42.60, 53.44)	54.24(48.61, 59.87)	44.85(39.40, 50.30)	43.38(38.65, 48.11)	-	17.99(14.65, 21.33)	21.19(16.88, 25.50)	11.23(9.24, 13.22)
Agriculture	40.60(34.45, 46.75)	62.18(53.85, 70.50)	59.36(53.32, 65.40)	46.03(40.62, 51.44)	55.08(48.62, 61.54)	51.77(39.54, 64.00)	-	25.20(23.37, 27.04)	37.44(32.11, 42.77)	15.10(13.44, 16.77)
Unskilled/manual worker	54.00(50.45, 57.55)	77.11(73.15, 81.06)	63.53(59.70, 67.36)	57.86(53.06, 62.65)	58.22(54.97, 61.48)	64.84(61.28, 68.40)	-	28.21(25.59, 30.83)	35.77(32.81, 38.74)	13.66(11.34, 15.98)

^⁺^ Information about men's occupations was not available in Dominican Republic,

‘0’ replaces a negative lower limit of confidence interval - - no observations in some categories

**Table 4:** Weighted prevalence estimates (%) and 95% CIs of smoking by social factors among women in 7 countries (North Africa, Central and West Asia).

	Albania	Armenia	Kyrgyz Republic	Tajikistan	Moldova	Ukraine	Jordan
**Type of domicile**							
Urban	7.91 (5.72, 10.10)	2.80 (1.78, 3.82)	5.24 (3.77, 6.71)	0.56 (0.15, 0.98)	13.61 (12.36, 14.85)	17.68 (15.75, 19.62)	19.63 (17.81, 21.46)
Rural	1.18 (0.71, 1.64)	0.14 (0, 0.35)	1.28 (0.75, 1.80)	0.07 (0, 0.15)	2.24 (1.42, 3.05)	7.67 (6.06, 9.28)	9.61 (7.70, 11.51)
**Age groups** (years)							
15–24	3.52 (2.03, 5.00)	0.31 (-0.03, 0.64)	2.78 (1.51, 4.04)	0.08 (0, 0.18)	7.26 (6.11, 8.40)	16.89(14.39, 19.40)	18.08 (13.78, 22.38)
25–34	7.10 (3.91, 10.30)	1.54 (0.53, 2.55)	2.36 (1.52, 3.19)	0.14 (0, 0.30)	8.92 (7.68, 10.16)	17.08 (14.60, 19.56)	15.87 (13.72, 18.02)
35–44	3.30 (2.53, 4.06)	2.58 (1.18, 3.97)	2.64 (1.64, 3.63)	0.40 (0.16, 0.63)	6.18 (5.07, 7.29)	14.49 (12.27, 16.71)	19.22 (16.65, 21.79)
45–49	2.94 (1.84, 4.05)	4.21 (1.95, 6.46)	3.97 (2.22, 5.73)	0.38 (0, 0.78)	5.37(3.79, 6.95)	7.77 (5.72, 9.82)	20.03 (16.01, 24.04)
**Marital status**							
Never in union	5.84 (3.00, 8.68)	1.35 (0.54, 2.16)	3.53 (2.07, 5.00)	0.07 (0, 0.18)	5.83 (4.62, 7.04)	17.56 (14.84, 20.28)	-
Married	2.79 (2.21, 3.36)	1.31 (0.66, 1.96)	1.60 (1.08, 2.12)	0.17 (0.04, 0.30)	4.92 (4.25, 5.59)	9.95 (8.49, 11.41)	17.07 (15.58, 18.55)
Cohabitation	-	-	-	-	23.20 (18.59, 27.82)	30.89 (22.99, 38.80)	-
Single	13.31 (9.05, 17.57)	7.77 (3.92, 11.62)	8.68 (6.28, 11.08)	1.05 (0.23, 1.88)	17.20 (14.37, 20.04)	22.28 (18.97, 25.58)	35.46 (27.49, 43.43)
**Education**							
No education	6.57 (0, 16.65)	-	-	-	25.41 (4.37, 46.45)	-	17.76 (7.17, 28.34)
primary	2.04 (1.48, 2.60)	1.60 (0.02, 3.17)	-	-	14.35 (-0.48, 29.18)	35.07 (12.08, 58.06)	20.25 (16.07, 24.44)
Secondary	3.04 (2.40, 3.69)	0.45 (0.08, 0.82)	1.70 (1.07, 2.33)	0.16 (0.02, 0.30)	5.96 (5.21, 6.71)	15.07 (13.03, 17.12)	19.35 (17.19, 21.50)
Higher	15.32 (8.29, 22.36)	2.62 (1.61, 3.63)	4.15 (3.20, 5.10)	0.47 (0.17, 0.77)	11.22 (9.74, 12.69)	14.60 (12.77, 16.43)	14.72 (12.66, 16.77)
**Wealth Index**							
Poorest	0.83 (0.16, 1.49)	0.21 (0, 0.54)	0.69 (0.14, 1.23)	-	1.08 (0.34, 1.83)	9.89 (7.25, 12.54)	13.88 (11.21, 16.55)
Poorer	1.12 (0.48, 1.76)	0.43 (0, 0.89)	0.68 (0.23, 1.14)	0.10 (0, 0.27)	2.43 (1.34, 3.52)	8.77 (6.93, 10.61)	14.89 (11.96, 17.81)
Middle	2.08 (1.21, 2.95)	1.43 (0.46, 2.40)	1.43 (0.72, 2.13)	0.01 (0, 0.03)	2.97 (2.09, 3.85)	16.83 (13.49, 20.18)	15.65 (13.26, 18.04)
Richer	5.03 (3.81, 6.24)	2.45 (1.18, 3.72)	3.37 (2.04, 4.70)	0.37 (0, 0.77)	8.46 (7.02, 9.90)	16.77 (14.06, 19.49)	17.59 (14.89, 20.29)
Richest	11.54 (7.89, 15.19)	4.17 (2.13, 6.21)	6.19 (4.31, 8.07)	0.44 (0, 0.71)	16.85 (15.03, 18.67)	18.94 (15.65, 22.22)	28.78 (24.83, 32.73)
**Occupation**							
Unemployed	2.97 (2.17, 3.78)	0.69 (0.32, 1.05)	1.33 (0.85, 1.81)	0.17 (0.01, 0.34)	5.40 (4.51, 6.29)	11.23 (9.40, 13.07)	17.54 (15.86, 19.24)
Professional	11.12 (6.85, 15.60)	4.93 (2.48, 7.39)	5.37 (3.67, 7.06)	0.53 (0.11, 0.95)	10.68 (9.19, 12.16)	16.91 (14.98, 18.85)	17.82 (13.79, 21.86)
Agriculture	0.89 (0, 1.84)	1.06 (0, 2.53)	-	-	1.54 (0.65, 2.44)	5.67 (1.97, 9.38)	20.19 (9.84, 30.55)
Unskilled/manual worker	5.34 (3.45, 7.22)	3.26 (1.35, 5.17)	8.42 (5.29, 11.56)	0.16 (0, 0.33)	9.89 (8.06, 11.73)	14.77 (11.74, 17.79)	31.89 (22.99, 40.80)

‘0’ replaces a negative lower limit of confidence interval, - no observations in this category

**Table 5:** Weighted prevalence estimates (%) and 95% CIs of smoking by social factors among women in 7 countries (Latin America, Caribbean and North Africa).

	Bolivia	Dominican Republic	Honduras	Peru⁺	Guyana	Haiti	Egypt
**Type of domicile**							
Urban	10.15 (9.23, 11.07)	6.07 (5.50, 6.64)	2.49 (2.04, 2.93)	6.31 (5.55, 7.07)	5.38 (4.16, 6.61)	2.58 (2.06, 3.11)	0.86 (0.59, 1.13)
Rural	5.75 (4.89, 6.62)	7.21 (6.53, 7.90)	0.85 (0.62, 1.09)	0.78 (0.53, 1.04)	2.42 (1.64, 3.21)	1.79 (1.35, 2.23)	0.42 (0.28, 0.55)
**Age groups** (years)							
15–24	7.32 (6.45, 8.18)	1.82 (1.43, 2.22)	1.86 (1.39, 2.33)	5.63 (4.77, 6.50)	1.54 (0.80, 2.28)	1.17 (0.81, 1.53)	0.34 (0.12, 0.57)
25–34	8.52 (7.49, 9.55)	5.60 (4.76, 6.43)	1.63 (1.23, 2.03)	4.96 (4.13, 5.79)	2.20 (1.33, 3.07)	1.96 (1.40, 2.53)	0.54 (0.35, 0.74)
35–44	9.29 (8.17, 10.40)	10.29 (9.25, 11.32)	1.32 (0.83, 1.81)	4.03 (3.13, 4.93)	5.70 (3.97, 7.42)	3.32 (2.44, 4.21)	0.86 (0.56, 1.16)
45–49	12.68 (10.73, 14.63)	16.48 (14.38, 18.57)	2.66 (1.70, 3.61)	4.81 (3.38, 6.24)	5.68 (3.35, 8.01)	5.54 (4.08, 7.00)	0.51 (0.19, 0.82)
**Marital status**							
Never in union	9.21 (8.09, 10.34)	1.53 (1.04, 2.03)	1.82 (1.31, 2.34)	6.62 (5.65, 7.59)	1.56 (0.78, 2.35)	0.46 (0.25, 0.67)	-
Married	7.35 (6.49, 8.21)	3.84 (2.94, 4.74)	0.83 (0.41, 1.25)	4.28 (2.95, 5.60)	2.14 (1.27, 3.01)	2.39 (1.91, 2.88)	0.54 (0.41, 0.67)
Cohabitation	7.35 (6.26, 8.43)	8.81 (8.06, 9.57)	1.68 (1.28, 2.08)	2.88 (2.28, 3.49)	4.69 (3.25, 6.12)	4.75 (3.49, 6.00)	-
Single	15.97 (13.53, 18.41)	9.16 (7.78, 10.54)	3.00 (2.27, 3.73)	7.67 (6.12, 9.23)	8.74 (5.67, 11.82)	4.97 (3.39, 6.54)	1.47 (0.59, 2.35)
**Education**							
No education	8.42 (6.18, 10.65)	19.40 (15.07, 23.73)	1.11 (0.37, 1.85)	0.90 (0.10, 1.70)	2.67 (0.02, 5.32)	4.34 (3.27, 5.40)	0.46 (0.28, 0.65)
primary	6.34 (5.58, 7.11)	9.71 (8.91, 10.50)	1.44 (1.12, 1.76)	0.98 (0.69, 1.26)	3.26 (1.97, 4.55)	2.07 (1.55, 2.59)	0.71 (0.33, 1.09)
Secondary	7.77 (6.86, 8.67)	3.99 (3.28, 4.69)	1.82 (1.44, 2.21)	4.44 (3.66, 5.23)	3.40 (2.65, 4.15)	1.61 (1.23, 2.00)	0.58 (0.37, 0.79)
Higher	15.96 (14.10, 17.83)	2.71 (1.87, 3.54)	3.57 (2.17, 4.96)	9.17 (7.89, 10.45)	2.61 (-0.66, 5.87)	1.38 (0.52, 2.24)	0.96 (0.34, 1.58)
**Wealth Index**							
Poorest	6.22 (5.01, 7.44)	10.05 (8.92, 11.19)	0.41 (0.26, 0.55)	1.00 (0.61, 1.39)	3.57 (2.05, 5.09)	2.21 (1.48, 2.95)	0.63 (0.31, 0.94)
Poorer	5.34 (4.24, 6.45)	8.27 (7.20, 9.34)	1.43 (0.92, 1.95)	0.81 (0.53, 1.10)	4.52 (2.78, 6.27)	2.02 (1.34, 2.69)	0.29 (0.09, 0.49)
Middle	6.74 (5.58, 7.91)	5.79 (4.87, 6.70)	1.99 (1.44, 2.54)	2.86 (2.19, 3.53)	2.43 (1.43, 3.44)	2.38 (1.66, 3.09)	0.46 (0.23, 0.69)
Richer	7.96 (6.80, 9.12)	4.56 (3.85, 5.27)	1.92 (1.33, 2.52)	5.12 (4.17, 6.07)	2.53 (1.31, 3.75)	2.16 (1.54, 2.77)	0.45 (0.21, 0.69)
Richest	14.86 (13.30, 16.41)	4.57 (3.52, 5.61)	2.50 (1.93, 3.08)	12.93 (11.03,14.82)	3.59 (1.84, 5.34)	2.09 (1.54, 2.63)	1.15 (0.67, 1.63)
**Occupation**							
Unemployed	6.12 (5.28, 6.97)	5.38 (4.81, 5.96)	1.30 (0.99, 1.62)	-	2.58 (1.78, 3.38)	1.41 (1.09, 1.72)	0.59 (0.44, 0.73)
Professional	12.15 (10.84, 13.47)	5.26 (4.50, 6.02)	2.09 (1.62, 2.56)	-	3.09 (1.24, 4.94)	2.62 (2.04, 3.20)	0.65 (0.23, 1.06)
Agriculture	7.28 (6.25, 8.30)	12.16 (10.41, 13.92)	0.92 (0.52, 1.32)	-	1.95 (0.49, 3.41)	4.11 (2.18, 6.03)	0.19 (0, 0.39)
Unskilled/manual worker	9.20 (7.65, 10.75)	8.63 (7.45, 10.75)	2.75 (1.96, 3.54)	-	8.28 (5.02, 11.54)	3.67 (1.91, 5.44)	1.24 (0.24, 2.25)

^⁺^ Information about women's occupations was not available in Peru,

‘0’ replaces a negative lower limit of confidence interval, - no observations in this category

**Table 6:** Binary logistic regression analyses for demographic and socio-economic factors associated with smoking among men in 10 countries (Central Asia, Latin America and Caribbean).

	**Albania**		**Armenia**		**Azerbaijan**		**Kyrgyz Republic**		**Ukraine**	
	**(aOR, 95%Cls)**	**p-value**	**(aOR, 95%Cls)**	**p-value**	**(aOR, 95%Cls)**	**p-value**	**(aOR, 95%Cls)**	**p-value**	**(aOR, 95%Cls)**	**p-value**
Age (years)	1.02(1.01,1.03)	0.001	1.04(1.02,1.06)	<0.001	1.02(1.01,1.04)	<0.001	1.07(1.06,1.09)	<0.001	1.02(1.01,1.03)	<0.001
Urban/ Rural	0.83(0.62,1.11)	0.199	0.98(0.62,1.55)	0.924	0.80(0.58,1.11)	0.182	0.92(0.65,1.31)	0.648	0.72(0.53,0.97)	0.034
Marital status	1.58(1.25,2.03)	<0.001	1.35(0.99,1.86)	0.06	1.90(1.43,2.51)	<0.001	1.51(1.15,1.97)	0.003	1.22(1.07,1.38)	0.003
Education	0.84(0.70,0.99)	0.041	0.84(0.68,1.04)	0.107	0.75(0.58,0.97)	0.027	1.30(1.03,1.63)	0.027	0.74(0.62,0.90)	0.002
Wealth Index	0.99(0.90,1.09)	0.856	1.12(0.95,1.32)	0.182	0.90(0.80,1.00)	0.05	1.25(1.13,1.38)	<0.001	0.86(0.76,0.97)	0.013
Occupation	1.32(1.22,1.45)	<0.001	1.45(1.26,1.65)	<0.001	1.35(1.22,1.48)	<0.001	1.46(1.30,1.67)	<0.001	1.39(1.28,1.49)	<0.001
	**Moldova**		**Dominican Republic⁺**		**Honduras**		**Guyana**		**Haiti**	
	**(aOR, 95%Cls)**	**p-value**	**(aOR, 95%Cls)**	**p-value**	**(aOR, 95%Cls)**	**p-value**	**(aOR, 95%Cls)**	**p-value**	**(aOR, 95%Cls)**	**p-value**
Age (years)	0.99(0.98,1.00)	0.012	1.04(1.03,1.04)	<0.001	0.99(0.98,0.99)	<0.001	1.03(1.02,1.04)	<0.001	1.02(1.02,1.03)	<0.001
Urban/Rural	0.75(0.57,0.99)	0.043	0.89(0.78,1.02)	0.09	0.73(0.60,0.89)	0.002	0.88(0.70,1.08)	0.217	0.92(0.64,1.31)	0.65
Marital status	1.68(1.43,1.97)	<0.001	1.46(1.36,1.55)	<0.001	1.48(1.35,1.62)	<0.001	1.46(1.32,1.63)	<0.001	1.65(1.49,1.82)	<0.001
Education	0.78(0.63,0.96)	0.02	0.76(0.70,0.83)	<0.001	0.93(0.81,1.07)	0.312	0.80(0.70,0.93)	0.003	0.78(0.68,0.90)	0.001
Wealth Index	0.82(0.73,0.90)	<0.001	0.88(0.84,0.93)	<0.001	0.84(0.79,0.91)	<0.001	0.72(0.66,0.78)	<0.001	0.96(0.85,1.08)	0.525
Occupation	1.17(1.09,1.25)	<0.001	-	-	1.21(1.08,1.35)	0.001	1.28(1.17,1.42)	<0.001	1.17(1.07,1.28)	0.001

^⁺^ Information about men's occupation was not available in Dominican Republic,

age was used a continuous variable whereas ‘urban’, ‘not in union’, ‘no education’, ‘poorest’, ‘unemployed’ were the reference categories

**Table 7:** Binary logistic regression analyses for demographic and socio-economic factors associated with smoking among WOMEN in 14 countries (Central Asia, North Africa, Latin America and the Caribbean).

	**Albania**		**Armenia**		**Kyrgyz Republic**		**Tajikistan**		**Ukraine**		**Egypt**		**Jordan**	
	(aOR, 95%Cls)	p-value	(aOR, 95%Cls)	p-value	(aOR, 95%Cls)	p-value	(aOR, 95%Cls)	p-value	(aOR, 95%Cls)	p-value	(aOR, 95%Cls)	p-value	(aOR, 95%Cls)	p-value
Age (years)	0.99 (0.97,1.00)	0.064	1.06 (1.03,1.11)	0.001	0.99 (0.97,1.01)	0.267	1.03 (0.98,1.08)	0.242	0.96 (0.95,0.97)	<0.001	1.02 (1.00,1.04)	0.108	1.00 (0.99,1.01)	0.918
Urban/ Rural	0.49 (0.29,0.17)	0.01	0.14 (0.03,0.57)	0.007	0.76 (0.32,1.79)	0.518	0.24 (0.05,1.34)	0.103	0.46 (0.33,0.64)	<0.001	0.61 (0.38,0.98)	0.04	0.50 (0.39,0.64)	<0.001
Marital status	1.22 (0.79,1.90)	0.366	1.63 (0.85,3.13)	0.138	1.30 (0.88,1.92)	0.191	3.25 (1.35,7.85)	0.009	1.42 (1.30,1.55)	<0.001	1.58 (1.12,2.27)	0.01	1.65 (1.42,1.93)	<0.001
Education	1.80 (1.22,2.69)	0.004	1.73 (0.90,3.39)	0.1	1.55 (1.01,2.39)	0.044	1.57 (0.62,4.01)	0.343	0.78 (0.64,0.95)	0.013	1.11 (0.84,1.46)	0.495	0.66 (0.58,0.76)	<0.001
Wealth Index	1.52 (1.25,1.86)	<0.001	1.51 (1.15,1.99)	0.003	1.58 (1.13,2.23)	0.008	1.36 (0.89,2.08)	0.158	1.12 (1.00,1.26)	0.049	1.06 (0.82,1.39)	0.641	1.32 (1.21,1.46)	<0.001
Occupation	1.17 (1.01,1.35)	0.042	1.31 (1.08,1.58)	0.005	1.62 (1.38,1.92)	<0.001	0.90 (0.50,1.60)	0.708	1.07 (0.97,1.19)	0.184	1.03 (0.75,1.42)	0.843	1.16 (1.01,1.32)	0.031
	**Moldova**		**Dominican Republic**		**Honduras**		**Guyana**		**Haiti**		**Bolivia**		**Peru⁺**	
	(aOR, 95%Cls)	p-value	(aOR, 95%Cls)	p-value	(aOR, 95%Cls)	p-value	(aOR, 95%Cls)	p-value	(aOR, 95%Cls)	p-value	(aOR, 95%Cls)	p-value	(aOR, 95%Cls)	p-value
Age (years)	0.97 (0.96,0.98)	<0.001	1.07 (1.06,1.08)	<0.001	0.99 (0.97,1.01)	0.333	1.04 (1.02,1.07)	<0.001	1.03 (1.02,1.05)	<0.001	1.02 (1.01,1.03)	<0.001	0.99 (0.97,1.00)	0.012
Urban/Rural	0.40 (0.25,0.65)	<0.001	0.86 (0.74,1.01)	0.066	0.44 (0.27,0.73)	0.002	0.39 (0.22,0.69)	0.001	0.50 (0.28,0.87)	0.015	1.03 (0.81,1.30)	0.833	0.68 (0.45,1.04)	0.073
Marital status	1.88 (1.68,2.10)	<0.001	1.32 (1.20,1.46)	<0.001	1.25 (1.07,1.45)	0.003	1.60 (1.30,1.97)	<0.001	1.63 (1.45,1.86)	<0.001	1.09 (1.01,1.19)	0.031	1.08 (0.98,1.21)	0.128
Education	0.90 (0.73,1.11)	0.289	0.63 (0.57,0.70)	<0.001	1.30 (0.97,1.73)	0.084	1.07 (0.71,1.62)	0.723	0.77 (0.64,0.93)	0.006	1.40 (1.28,1.55)	<0.001	1.48 (1.25,1.73)	<0.001
Wealth Index	1.68 (1.43,1.97)	<0.001	0.87 (0.80,0.95)	0.002	1.07 (0.92,1.26)	0.339	0.84 (0.69,1.02)	0.074	0.89 (0.73,1.08)	0.255	1.23 (1.13,1.34)	<0.001	2.01 (1.77,2.27)	<0.001
Occupation	1.07 (0.97,1.20)	0.149	1.05 (1.00,1.12)	0.065	1.20 (1.05,1.36)	0.006	1.22 (0.97,1.54)	0.092	1.17 (0.99,1.39)	0.071	1.14 (1.07,1.22)	<0.001	-	-

^⁺^ Information about women's occupations was not available in Peru,

age was used a continuous variable whereas ‘urban’, ‘not in union’, ‘no education’, ‘poorest’, ‘unemployed’ were the reference categories

### Age distribution

The prevalence of male smoking was higher in older age groups, i.e. >45 years in Armenia, Kyrgyz Republic, Ukraine, Dominican Republic and Haiti but in Honduras it was nearly the same in all age groups. The age differential was highest in the Kyrgyz Republic (15–24 years vs. >45 years was 16.4% vs. 67.5%) (Table 3). The prevalence of female smoking was also higher in older age groups in Armenia and Bolivia while in Dominican Republic, the age differential was highest (15–24 years vs. 45–49 years was 1.8% vs. 16.5%) (Tables 4 and 5). Men's age was associated with smoking in all countries, but women's age was not associated with smoking in Armenia, Ukraine, Moldova, Dominican Republic, Guyana, Haiti and Bolivia. Older men and women were more likely to smoke in most countries except for Ukraine and Jordan (for women only) (Tables 6 and 7).

### Distribution by wealth and education

Male smoking was slightly higher among the rich in most countries. However, in Moldova, Ukraine, Dominican Republic, Honduras and Guyana male smoking was the highest among the poorest (Table 3). In contrast, female smoking was highest among the richest in most of the countries except Guyana, Haiti and Dominican Republic (Tables 4 and 5). Compared to the richest, poorest men were more likely to smoke in Ukraine, Moldova, Dominican Republic, Honduras and Guyana (Table 6). Wealthier women were more likely to smoke in Albania, Armenia, Kyrgyz, Jordan, Moldova, Dominican Republic, and Bolivia (Table 7).

Both male and female smoking rates were lower among higher educated in Moldova, Dominican Republic and Haiti but in all other countries, male and female smoking rates were higher among the higher educated (Tables 3, 4 and 5). In most countries, smoking was associated with men's education, i.e. educated men were more likely to smoke in all countries except Armenia (aOR 0.84, 95% CI 0.68, 1.04) and Honduras (aOR 0.93, 95% CI 0.81, 1.07). Smoking was associated with women's education in Albania, Kyrgyz, Ukraine, Jordan, Dominican Republic, Haiti, Bolivia and Peru (Tables 6 and 7).

### Distribution by marital status and occupation

In general, smoking prevalence was higher among single men and women in most of the countries. In Moldova and Ukraine, smoking was highest among women living with a cohabiting partner (Tables 3, 4 and 5). Single men were more likely to be smokers in all the countries except Armenia, whereas single women were more likely to smoke in all except Albania, Armenia, Kyrgyz Republic and Peru (Tables 6 and 7). Male smoking was higher and highest among agriculturists and unskilled workers respectively in most countries (Table 3) whereas female smoking was highest among professionals in most countries except Dominican Republic and Guyana (Tables 4 and 5). Smoking was associated with men's occupation in all countries, but with women's occupation in five countries only (Table 7). Compared to unemployed, male unskilled workers and female unskilled workers or professionals were more likely to smoke (Tables 6 and 7).

## Discussion

Our study provides country-level prevalence estimates of current smoking and SLT use and highlights that the prevalence was higher among men and cigarette smoking was very common. In most countries, both sexes, the prevalence of SLT use was <5%. Smoking among both men and women showed within-country and cross-country variations by social factors. Male smoking was highest among the Central and West Asian countries, while female smoking was higher in Ukraine (15%) and Jordan (18%). In most countries, the prevalence of SLT among both men and women was lower than 5%, except in Kyrgyz, Haiti and Bolivia, contrary to India, Nepal, Pakistan and Bangladesh where SLT use prevalence among men exceeded 20%[6]. Among SLT users, snuff (snus) was the commonest product consumed by both men and women in Haiti and Dominican Republic.

Current smoking rates for most countries were comparable to daily smoking rates reported by Ng et al. in Global Burden of Disease (GBD) study except for Bolivia, Guyana, and Haiti (a difference of >5%). These differences may have occurred because the GBD study used comprehensive data sources, and robust statistical methods for estimating pooled prevalence rates of daily smoking defined as smoking any type of tobacco product at least once per day [4]. The reasons for the difference between our estimates and those of GBD in some countries are discussed in detail in previous studies [6,20]. Current smoking rates in our study were comparable to those reported from former Soviet Union countries, namely Armenia, Kyrgyz Republic, Moldova and Ukraine [25]. Smoking rates for Ukraine were fairly consistent, albeit different survey years and slightly different definition for current smoking i.e. daily and occasional smokers used in GATS [26] and WHS [27]. Higher male smoking rates in Central Asian countries may have been due to economic transition of the former Soviet Union, privatization of tobacco industries and cultural acceptance of male smoking, which has a long history of being a leisure time activity in the former Soviet Union [28]. In our study, male and female smoking rates for Latin American and Caribbean countries were lower than the WHO estimates (1989 to 1992)[29] probably attributable to the impact of smoke-free policies implemented in all Latin American and the Caribbean countries [30]. In a four-country survey (2001–2004), prevalence of current smoking defined as having smoked any number of cigarettes in the last 30 days among Honduran men was 29.8%[14].

Smoking rates among men was much higher than women in all countries which was similar to the results of a DHS-based study from Sub Saharan Africa [20,31], South and Southeast Asian countries [6]. However, SLT use rates among women in this study were <1.0%, while the SLTuse rates were much higher in India, Cambodia, Nepal, Madagascar and Lesotho [6,20]. Increase in smoking rates by age was significant in most countries similar to results of DHS-based studies [6,20,31], GATS [26] and WHS [27]. Smoking rates were higher among higher educated men and women in most countries are contrary to the pattern observed in previous surveys [6,26,31]. However, a study based on WHS has reported that higher educated women aged 45 years and above in Eastern European and Latin American countries had higher smoking rates [32]. Similar to our previous studies [6,20], there was a gradient in male smoking rates by education, i.e. lower smoking rates among the highest educated men and vice versa in most countries. However, in most countries wealthier women had higher smoking rates than the poorer women. Similar results were reported for smoking rates among women from middle income countries in WHS [27]. Women in these countries who may also be earning have personal economic freedom to purchase cigarettes or higher education and financial independence improves their social status and hence an autonomy to emulate their male counterparts’ life style. The pattern of higher smoking rates among rich and educated in some Latin American and Central Asian countries is comparable to the pattern observed in developed countries at the beginning of the smoking epidemic during the early twentieth century [33]. Higher smoking rates in rural population has been reported in previous studies [6,20,31], but we found significant urban-rural differentials in only a few countries similar to the results of GATS [26] and low income countries in WHS [27]. One explanation for higher smoking rates in rural populations could be that uneducated and poor is likely to be living in rural areas. Similar to our previous studies [6,20], the association of smoking with being single was not consistent across the countries. A plausible explanation for higher smoking rates among single men and women could be loneliness [34]. Male smokers were likely to be unskilled workers and agriculturists similar to results from Sub Saharan Africa [20,31] and United States of America [35]. Higher female smoking rates among professional women could also be explained by higher education and income, reflecting the social standing of an individual [36] which is known to affect the individual’s health and health behavior [37].

Our results should be interpreted with caution considering the following limitations. The prevalence rates of tobacco use were based on self-report, and are likely to be underestimated due to reporting bias [38]. However, the DHS survey did not verify self-reported tobacco use by measuring bio-markers such as urinary cotinine levels. Limited questions asked about tobacco use in DHS allowed us to classify 'current smoking' or 'current SLT use' only, whereas WHS [27] and GATS [26] included a detailed questionnaire on tobacco use and provided estimates of 'never user' and 'former user'. We assessed the association of smoking with only some socio-demographic factors. However, smoking behavior has complex relationships with social, personal and family factors which could not be studied from DHS data [39]. Moreover, the temporality of the association between smoking and social factors cannot be ascertained in cross-sectional DHSs since it is not possible to ascertain if there was sufficient time elapsed between the social factors and onset of smoking behavior. Prevalence estimates of smoking and/or SLT use could not be obtained for some countries because either tobacco use questions (smoking and/or SLT use) were not included in the DHS and in some countries only women were sampled or only women/men were asked about tobacco use.

Albeit the limitations, our study provided country-level weighted prevalence estimates for, North African, Central and Western Asian, Latin American, and Caribbean countries, and make cross-country comparisons similar to previous studies on tobacco use [6,20,31]. Moreover, prevalence estimates based on larger samples of men and women than the WHS [27] or GATS [26] may be more precise. DHSs may include more countries and cover both sexes with the more detailed tobacco use questions to provide estimates of 'never user', 'former user' and intentions to quit and quit attempts. Since DHSs cover more than 85 LMICs, and are repeated at regular intervals, monitoring of the tobacco use epidemic by time trend analyses could be possible.

## Conclusion

Our results confirm that male current smoking rates are higher in Central and West Asian countries, but SLT use is low in most countries. The social pattern of smoking varied across the countries, but the social pattern of smoking by wealth and education was contrasting between men and women in most countries. Our findings highlight that country-level analysis on the type of tobacco use and social distribution would be useful to identify the vulnerable population subgroups and help in formulating context-specific tobacco control policies and strategies.

## Supporting Information

S1 Fig Proportional distribution of tobacco products consumed by male tobacco users in 10 countries. We did not present the percentage of respondents using multiple tobacco products since the numbers were very small.
(TIF)

S2 Fig Proportional distribution of tobacco products consumed by female tobacco users in 15 countries. We did not present the percentage of respondents using multiple tobacco products since the number were very small.
(TIF)

S1 Table Descriptives (number and percentage) of social factors among men in 10 countries (Central Asia, Latin America and Caribbean). (DOCX)

S2 Table Descriptives (number and percentage) of social factors among women in 8 countries (Central Asia and North Africa). (DOCX)

S3 Table Descriptives (number and percentage) of social factors among women in 6 countries (Latin America and Caribbean). (DOCX)
