# Supplementary figures and images for: Prevalence and Social Determinants of Smoking in 15 Countries from North Africa, Central and Western Asia, Latin America and Caribbean: Secondary Data Analyses of Demographic and Health Surveys
Source: PLoS One. 2015 Jul 1;10(7):e0130104. doi: 10.1371/journal.pone.0130104 (PMC4488463; doi:10.1371/journal.pone.0130104)

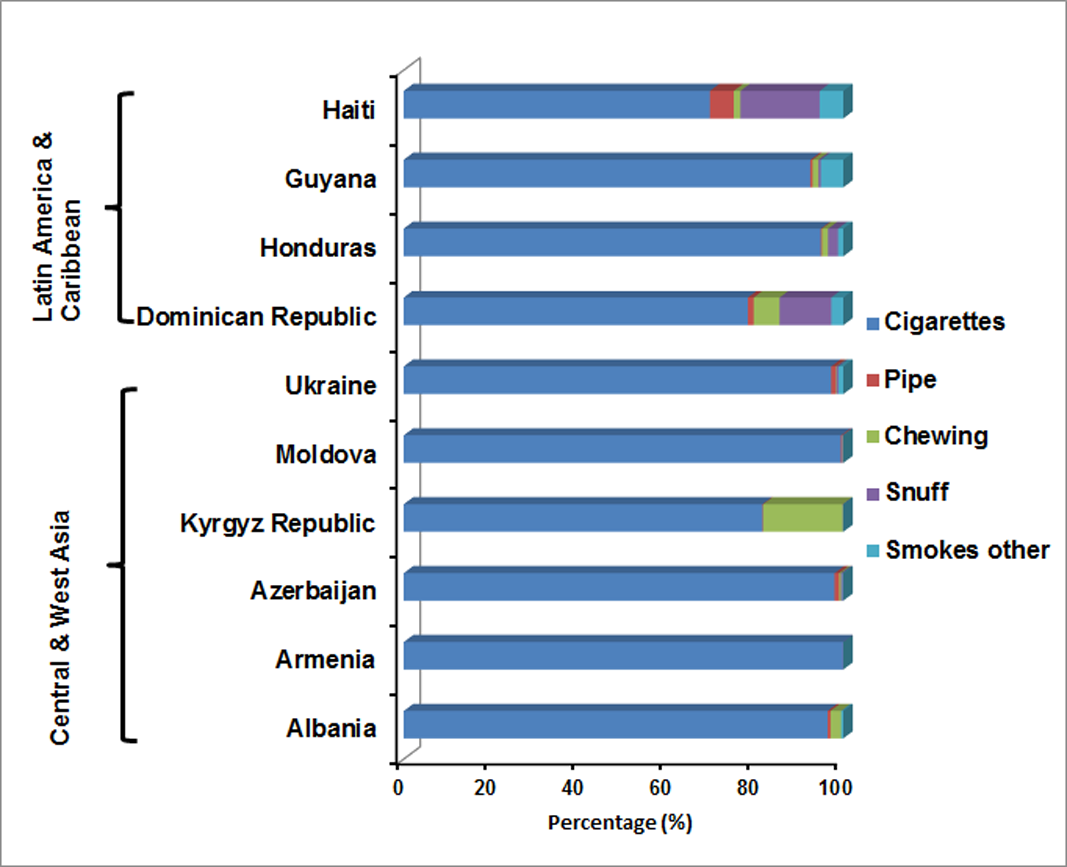

Supplement: S1 Fig — We did not present the percentage of respondents using multiple tobacco products since the numbers were very small. (TIF) [file pone.0130104.s001.tif]

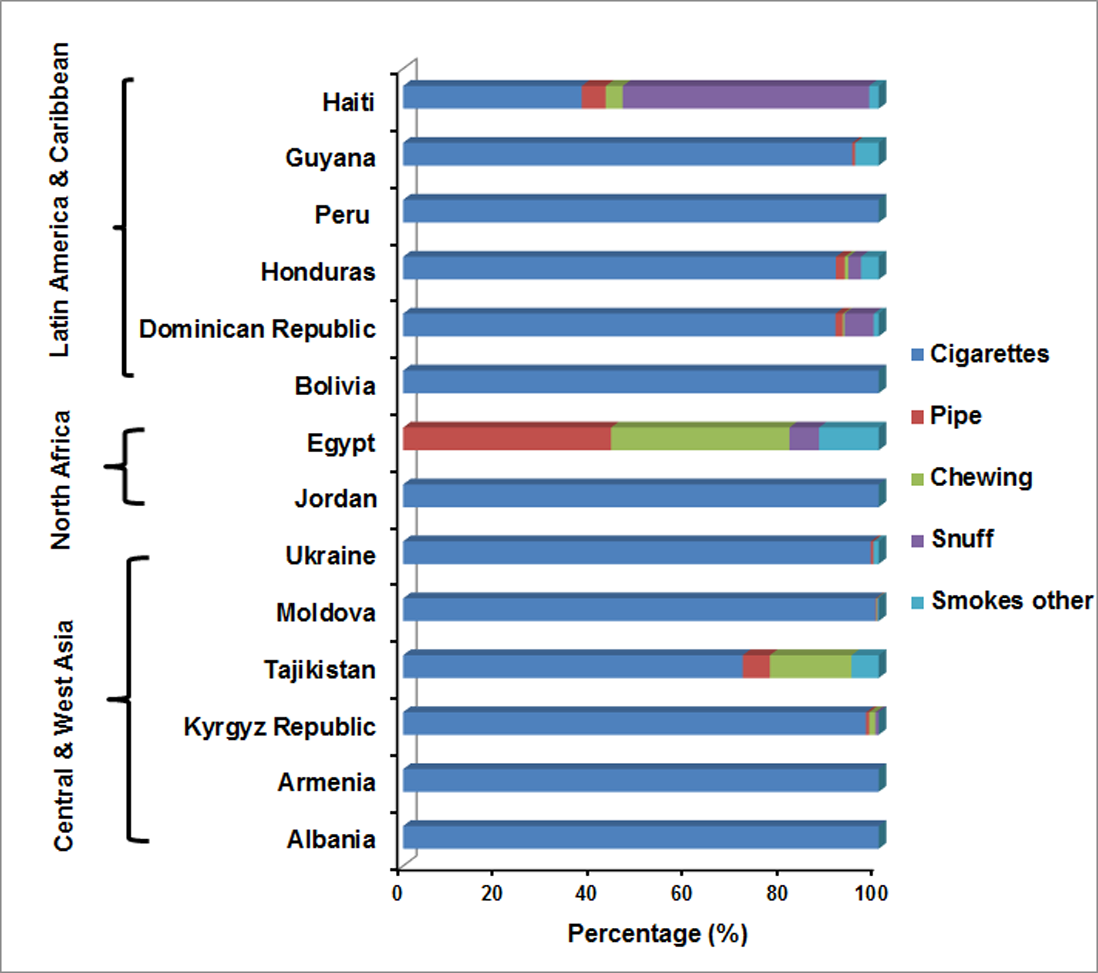

Supplement: S2 Fig — We did not present the percentage of respondents using multiple tobacco products since the number were very small. (TIF) [file pone.0130104.s002.tif]
