# Supplementary material for: Prevalence and Social Determinants of Smoking in 15 Countries from North Africa, Central and Western Asia, Latin America and Caribbean: Secondary Data Analyses of Demographic and Health Surveys
Source: PLoS One. 2015 Jul 1;10(7):e0130104. doi: 10.1371/journal.pone.0130104 (PMC4488463; doi:10.1371/journal.pone.0130104)
Supplement: S1 Table — (DOCX) [file pone.0130104.s003.docx]

Web Appendix Table S1 Descriptives (number and percentage) of social factors among MEN in 10 countries (Central Asia, Latin America and Caribbean)

|  | Albania  (N=3013) | Armenia  (N=1584) | Azerbaijan  (N=2558) | Kyrgyz Republic  (N=2413) | Moldova  (N=2508) | Ukraine  (N=3178) | Dominican Republic (N=27975) | Honduras  (N=7120) | Guyana  (N=3522) | Haiti  (N=9493) |
| --- | --- | --- | --- | --- | --- | --- | --- | --- | --- | --- |
| Median age (q1,q3) | 33 (20, 43) | 29 (22, 39) | 34 (23, 44) | 29 (22, 39) | 37 (23, 48) | 32 (23, 41) | 30 (21, 43) | 32 (23, 42) | 30 (21, 39) | 28 (20, 40) |
| Age groups | | | | | | | | | | |
| 15-24 | 1055 (35.0) | 536 (33.8) | 751 (29.4) | 822 (34.1) | 698 (27.8) | 910 (28.6) | 9874 (35.3) | 2065 (29.0) | 1217 (34.6) | 3834 (40.4) |
| 25-34 | 549 (18.2) | 505 (31.9) | 568 (22.2) | 710 (29.4) | 469 (18.7) | 903 (28.4) | 6516 (23.3) | 1985 (27.9) | 947 (26.9) | 2274 (24.0) |
| 35-44 | 859 (28.5) | 334 (21.1) | 625 (24.4) | 617 (25.6) | 489 (19.5) | 854 (26.9) | 5660 (20.2) | 1555 (21.8) | 945 (26.8) | 1606 (16.9) |
| ≥45 | 550 (18.3) | 209 (13.2) | 614 (24.0) | 264 (10.9) | 852 (34.0) | 511 (16.1) | 5925 (21.2) | 1515 (21.3) | 413 (11.7) | 1779 (18.7) |
| Type of domicile | | | | | | | | | | |
| Urban | 1655 (54.9) | 1063 (67.1) | 1357 (53) | 690 (28.6) | 1417 (56.5) | 1993 (62.7) | 15669 (56.0) | 2728 (38.3) | 1013 (28.8) | 3807 (40.1) |
| Rural | 1358 (45.1) | 521 (32.9) | 1201 (47) | 1723 (71.4) | 1091 (43.5) | 1185 (37.3) | 12306 (44.0) | 4392 (61.7) | 2509 (71.2) | 5686 (59.9) |
| Marital status | | | | | | | | | | |
| Not in union | 1310 (43.5) | 701 (44.3) | 855 (33.4) | 853 (35.4) | 745 (29.7) | 1058 (33.3) | 10329 (36.9) | 1768 (24.8) | 1377 (39.1) | 4596 (48.4) |
| Married | 1650 (54.8) | 811 (51.2) | 1653 (64.6) | 1465 (60.7) | 1561 (62.2) | 1645 (51.8) | 3388 (12.1) | 1999 (28.1) | 1159 (32.9) | 3895 (41.0) |
| Cohabiting | - | - | - | - | 83 (3.3) | 158 (5.0) | 10410 (37.2) | 2761 (38.8) | 725 (20.6) | 439 (4.6) |
| Single | 53 (1.8) | 72 (4.5) | 50 (2.0) | 95 (3.9) | 119 (4.7) | 317 (10.0) | 3693 (13.2) | 592 (8.3) | 261 (7.4) | 563 (5.9) |
| Education | | | | | | | | | | |
| No education | 20 (0.7) | - | 13 (0.5) | 1 (0.0) | 3 (0.1) | 2 (0.1) | 1654 (5.9) | 483 (6.8) | 56 (1.6) | 1325 (14.0) |
| primary | 1176 (39.0) | 182 (11.5) | 18 (0.7) | 7 (0.3) | 11 (0.4) | 3 (0.1) | 14173 (50.7) | 4400 (61.8) | 741 (21.0) | 3767 (39.7) |
| Secondary | 1460 (48.5) | 657 (41.5) | 2081 (81.4) | 1557 (64.5) | 2030 (80.9) | 1694 (53.3) | 9180 (32.8) | 1849 (26.0) | 2451 (69.6) | 3811 (40.1) |
| Higher | 357 (11.8) | 745 (47.0) | 446 (17.4) | 848 (35.1) | 464 (18.5) | 1479 (46.5) | 2968 (10.6) | 388 (5.4) | 274 (7.8) | 590 (6.2) |
| Wealth Index | | | | | | | | | | |
| Poorest | 513 (17.0) | 312 (19.7) | 519 (20.3) | 562 (23.3) | 342 (13.6) | 508 (16.0) | 9106 (32.6) | 1828 (25.7) | 900 (25.6) | 2143 (22.6) |
| Poorer | 498 (16.5) | 314 (19.8) | 526 (20.6) | 550 (22.8) | 397 (15.8) | 783 (24.6) | 6002 (21.5) | 1614 (22.7) | 675 (19.2) | 1940 (20.4) |
| Middle | 558 (18.5) | 346 (21.8) | 575 (22.5) | 475 (19.7) | 428 (17.1) | 661 (20.8) | 5197 (18.6) | 1442 (20.3) | 667 (18.9) | 2027 (21.4) |
| Richer | 736 (24.4) | 362 (22.9) | 515 (20.1) | 411 (17.0) | 656 (26.2) | 549 (17.3) | 4378 (15.6) | 1183 (16.6) | 695 (19.7) | 1742 (18.4) |
| Richest | 708 (23.5) | 250 (15.8) | 423 (16.5) | 415 (17.2) | 685 (27.3) | 677 (21.3) | 3292 (11.8) | 1053 (14.8) | 585 (16.6) | 1641 (17.3) |
| Occupation | | | | | | | | | | |
| Unemployed | 776 (25.8) | 429 (27.1) | 773 (30.2) | 518 (21.5) | 851 (33.9) | 641 (20.2) | - | 268 (3.8) | 437 (12.4) | 2127 (22.4) |
| Professional | 604 (20) | 274 (17.3) | 572 (22.4) | 450 (18.6) | 348 (13.9) | 821 (25.8) | - | 1167 (16.4) | 506 (14.4) | 1831 (19.3) |
| Agriculture | 409 (13.6) | 158 (10.0) | 292 (11.4) | 606 (25.1) | 303 (12.1) | 68 (2.1) | - | 3327 (46.7) | 641 (18.2) | 3944 (41.5) |
| Unskilled | 1224 (40.6) | 723 (45.6) | 921 (36.0) | 839 (34.8) | 1006 (40.1) | 1648 (51.9) | - | 2358 (33.1) | 1938 (55.0) | 1591 (16.8) |
| Smokers | 1319 (43.8) | 980 (61.9) | 1223 (47.8) | 1029 (42.6) | 1261 (50.3) | 1677 (52.8) | 3310 (11.8) | 1690 (23.7) | 1068 (30.3) | 1100 (11.6) |
| SLT users | 33 (1.1) | 1 (0.1) | 10(0.4) | 227 (9.4) | 3 (0.1) | 10 (0.3) | 723 (2.6) | 64 (0.9) | 21 (0.6) | 298 (3.1) |
