# Supplementary material for: Prevalence and Social Determinants of Smoking in 15 Countries from North Africa, Central and Western Asia, Latin America and Caribbean: Secondary Data Analyses of Demographic and Health Surveys
Source: PLoS One. 2015 Jul 1;10(7):e0130104. doi: 10.1371/journal.pone.0130104 (PMC4488463; doi:10.1371/journal.pone.0130104)
Supplement: S2 Table — (DOCX) [file pone.0130104.s004.docx]

Web appendix Table S2 Descriptives (number and percentage) of social factors among WOMEN in 8 countries (Central Asia and North Africa)

|  | Albania  (N=7584) | Armenia  (N=5922) | Kyrgyz Republic  (N=8208) | Tajikistan  (N=9656) | Moldova  (N=7440) | Ukraine  (N=6841) | Egypt  (N=19474) | Jordan  (N=11352) |
| --- | --- | --- | --- | --- | --- | --- | --- | --- |
| Median age (q1,q3) | 33 (22,41) | 30 (22,41) | 28 (21,38) | 27 (21,37) | 31 (21,41) | 33 (24,41) | 33 (26,40) | 34 (28,41) |
| Age groups | | | | | | | | |
| 15-24 | 2471 (32.6) | 1898 (32.0) | 3105 (37.8) | 3901 (40.4) | 2548 (34.2) | 1807 (26.4) | 3866 (19.9) | 1429 (12.6) |
| 25-34 | 1688 (22.3) | 1728 (29.2) | 2328 (28.4) | 2739 (28.4) | 1882 (25.3) | 1955 (28.6) | 6969 (35.8) | 4279 (37.7) |
| 35-44 | 2364 (31.2) | 1401 (23.7) | 1874 (22.8) | 2102 (21.8) | 1860 (25.0) | 1997 (29.2) | 6013 (30.9) | 4163 (36.7) |
| 45-49/59 | 1061 (14.0) | 895 (15.1) | 901 (11.0) | 914 (9.5) | 1150 (15.5) | 1082 (15.8) | 2626 (13.5) | 1481 (13.0) |
| Type of domicile | | | | | | | | |
| Urban | 3846 (50.7) | 3966 (67.0) | 2732 (33.3) | 3408 (35.3) | 4301 (57.8) | 4291 (62.7) | 8095 (41.6) | 8034 (70.8) |
| Rural | 3738 (49.3) | 1956 (33.0) | 5476 (66.7) | 6248 (64.7) | 3139 (42.2) | 2550 (37.3) | 11379 (58.4) | 3318 (29.2) |
| Marital status | | | | | | | | |
| Not in union | 2412 (31.8) | 1831 (30.9) | 2101 (25.6) | 2723 (28.2) | 1884 (25.3) | 1520 (22.2) | - | - |
| Married | 4878 (64.3) | 3679 (62.1) | 5452 (66.4) | 6364 (65.9) | 4486 (60.3) | 3882 (56.7) | 18134 (93.1) | 10746 (94.7) |
| Cohabiting | 294 (3.9) | 412 (7.0) | 655 (8.0) | 569 (5.9) | 406 (5.5) | 313 (4.6) | - | - |
| Single | - | - | - | - | 664 (8.9) | 1126 (16.5) | 1340 (6.9) | 606 (5.3) |
| Education | | | | | | | | |
| No education | 32 (0.4) | 4 (0.1) | 7 (0.1) | 155 (1.6) | 19 (0.3) | 2 (0.0) | 6934 (35.6) | 408 (3.6) |
| primary | 3674 (48.4) | 360 (6.1) | 29 (0.4) | 330 (3.4) | 30 (0.4) | 10 (0.1) | 3064 (15.7) | 981 (8.6) |
| Secondary | 2904 (38.3) | 2210 (37.3) | 4735 (57.7) | 7468 (77.3) | 5704 (76.7) | 2931 (42.8) | 7674 (39.4) | 6409 (56.5) |
| Higher | 974 (12.8) | 3348 (56.5) | 3437 (41.9) | 1703 (17.6) | 1686 (22.7) | 3898 (57.0) | 1802 (9.3) | 3554 (31.3) |
| Wealth Index | | | | | | | | |
| Poorest | 1622 (21.4) | 1066 (18.0) | 1666 (20.3) | 1616 (16.7) | 948 (12.7) | 1004 (14.7) | 4227 (21.7) | 2695 (23.7) |
| Poorer | 1314 (17.3) | 1305 (22.0) | 1653 (20.1) | 1625 (16.8) | 1004 (13.5) | 1711 (25.0) | 3882 (19.9) | 2896 (25.5) |
| Middle | 1341 (17.7) | 1332 (22.5) | 1641 (20.0) | 1736 (18.0) | 1376 (18.5) | 1391 (20.3) | 3669 (18.8) | 2601 (22.9) |
| Richer | 1737 (22.9) | 1284 (21.7) | 1570 (19.1) | 1930 (20.0) | 1923 (25.8) | 1266 (18.5) | 3791 (19.5) | 2050 (18.1) |
| Richest | 1570 (20.7) | 935 (15.8) | 1678 (20.4) | 2749 (28.5) | 2189 (29.4) | 1469 (21.5) | 3905 (20.1) | 1110 (9.8) |
| Occupation |  |  |  |  |  |  |  |  |
| Unemployed | 4698 (61.9) | 3906 (66.0) | 5730 (69.8) | 6829 (70.7) | 3232 (43.4) | 2007 (29.3) | 15176 (77.9) | 9344 (82.3) |
| Professional | 1273 (16.8) | 1100 (18.6) | 1648 (20.1) | 854 (8.8) | 2309 (31.0) | 3419 (50.0) | 2373 (12.2) | 1618 (14.3) |
| Agriculture | 970 (12.8) | 283 (4.8) | 187 (2.3) | 201 (2.1) | 674 (9.1) | 131 (1.9) | 1156 (5.9) | 165 (1.5) |
| Unskilled | 643 (8.5) | 633 (10.7) | 643 (7.8) | 1772 (18.4) | 1225 (16.5) | 1284 (18.8) | 769 (3.9) | 225 (2.0) |
| Smokers | 293 (3.9) | 60 (1.0) | 156 (1.9) | 26 (0.3) | 624 (8.4) | 870 (12.7) | 116 (0.6) | 1546 (13.6) |
| SLT users | - | - | 3 (0.0) | 6 (0.1) | 2 (0.0) | - | 7 (0.0) | - |
