# Supplementary material for: Prevalence and Social Determinants of Smoking in 15 Countries from North Africa, Central and Western Asia, Latin America and Caribbean: Secondary Data Analyses of Demographic and Health Surveys
Source: PLoS One. 2015 Jul 1;10(7):e0130104. doi: 10.1371/journal.pone.0130104 (PMC4488463; doi:10.1371/journal.pone.0130104)
Supplement: S3 Table — (DOCX) [file pone.0130104.s005.docx]

Web appendix Table S3 Descriptives (number and percentage) of social factors among WOMEN in 6 countries (Latin America and Caribbean)

|  | Dominican Republic  (N=27195) | Honduras  (N=22757) | Guyana  (N=4996) | Haiti  (N=14287) | Bolivia  (N=16939) | Peru  (N=23888) |
| --- | --- | --- | --- | --- | --- | --- |
| Median age (q1,q3) | 29 (21,38) | 27 (20,36) | 30 (21,39) | 26 (20,36) | 28 (21,38) | 30 (22,39) |
| Age groups | | | | | | |
| 15-24 | 10204 (37.5) | 9347 (41.1) | 1791 (35.8) | 6272 (43.9) | 6335 (37.4) | 8078 (33.8) |
| 25-34 | 7644 (28.1) | 6736 (29.6) | 1319 (26.4) | 4102 (28.7) | 5075 (30) | 6869 (28.8) |
| 35-44 | 6738 (24.8) | 4836 (21.3) | 1321 (26.4) | 2727 (19.1) | 3929 (23.2) | 6330 (26.5) |
| 45-49/59 | 2609 (9.6) | 1838 (8.1) | 565 (11.3) | 1186 (8.3) | 1600 (9.4) | 2611 (10.9) |
| Type of domicile | | | | | | |
| Urban | 16376 (60.2) | 9534(41.9) | 1420 (28.4) | 6321 (44.2) | 10905 (64.4) | 16006 (67.0) |
| Rural | 10819 (39.8) | 13223 (58.1) | 3576 (71.6) | 7966 (55.8) | 6034(35.6) | 7882 (33.0) |
| Marital status | | | | | | |
| Not in union | 6242 (23.0) | 6355 (27.9) | 1512 (30.3) | 5246 (36.7) | 5391 (31.8) | 7308 (30.6) |
| Married | 3566 (13.1) | 5071 (22.3) | 1803 (36.1) | 6295 (44.1) | 6233 (36.8) | 5517 (23.1) |
| Cohabiting | 12306 (45.3) | 8107 (35.6) | 1203 (24.1) | 1554 (10.9) | 3955 (23.3) | 8718 (36.5) |
| Single | 5081 (18.7) | 3224 (14.2) | 478 (9.6) | 1192 (8.3) | 1360 (8) | 2345 (9.8) |
| Education | | | | | | |
| No education | 1313 (4.8) | 1088 (4.8) | 81(1.6) | 2282 (16.0) | 749 (4.4) | 695 (2.9) |
| primary | 11129 (40.9) | 12425 (54.6) | 1042 (20.9) | 5551 (38.9) | 6846 (40.4) | 6130 (25.7) |
| Secondary | 10148 (37.3) | 7837 (34.4) | 3500 (70.1) | 5807 (40.6) | 6088 (35.9) | 10822 (45.3) |
| Higher | 4605 (16.9) | 1407 (6.2) | 373 (7.5) | 647 (4.5) | 3256 (19.2) | 6241 (26.1) |
| Wealth Index | | | | | | |
| Poorest | 6768 (24.9) | 5209(22.9) | 1254 (25.1) | 2802 (19.6) | 2822 (16.7) | 4779 (20.0) |
| Poorer | 5917(21.8) | 4977(21.9) | 899(18.0) | 2655 (18.6) | 2937 (17.3) | 5578 (23.4) |
| Middle | 5679(20.9) | 4529(19.9) | 936(18.7) | 3079 (21.6) | 3377 (19.9) | 5360 (22.4) |
| Richer | 4917(18.1) | 4211(18.5) | 984(19.7) | 2836 (19.9) | 3732 (22) | 4679 (19.6) |
| Richest | 3914(14.4) | 3831(16.8) | 923(18.5) | 2915 (20.4) | 4071 (24) | 3492 (14.6) |
| Occupation | | | | | | |
| Unemployed | 14415 (53.0) | 10531 (46.3) | 3054 (61.1) | 6911 (48.4) | 5179 (30.6) | - |
| Professional | 6551 (24.1) | 5135 (22.6) | 1027 (20.6) | 5791 (40.5) | 5153 (30.4) | - |
| Agriculture | 2287 (8.4) | 3184 (14.0) | 370 (7.4) | 764 (5.3) | 4457 (26.3) | - |
| Unskilled | 3942 (14.5) | 3907 (17.2) | 545 (10.9) | 821 (5.7) | 2150 (12.7) | - |
| Smokers | 1770 (6.5) | 353 (1.6) | 157 (3.1) | 311 (2.2) | 1447 (8.5) | 863 (3.6) |
| SLT users | 125 (0.5) | 13 (0.1) | - | 414 (2.9) | - | - |
